# 2-arachidonoylglycerol signaling impairs short-term fear extinction

**DOI:** 10.1038/tp.2016.26

**Published:** 2016-03-01

**Authors:** N D Hartley, O Gunduz-Cinar, L Halladay, O Bukalo, A Holmes, S Patel

**Affiliations:** 1 Department of Psychiatry, Vanderbilt University School of Medicine, Nashville, TN, USA; 2 Vanderbilt Brain Institute, Vanderbilt University School of Medicine, Nashville, TN, USA; 3 Laboratory of Behavioral and Genomic Neuroscience, National Institute on Alcoholism and Alcohol Abuse, National Institutes of Health, Bethesda, MD, USA; 4 Department of Molecular Physiology and Biophysics, Vanderbilt University School of Medicine, Nashville, TN, USA

## Abstract

Impairments in fear extinction are thought to be central to the psychopathology of posttraumatic stress disorder, and endocannabinoid (eCB) signaling has been strongly implicated in extinction learning. Here we utilized the monoacylglycerol lipase inhibitor JZL184 to selectively augment brain 2-AG levels combined with an auditory cue fear-conditioning paradigm to test the hypothesis that 2-AG-mediated eCB signaling modulates short-term fear extinction learning in mice. We show that systemic JZL184 impairs short-term extinction learning in a CB1 receptor-dependent manner without affecting non-specific freezing behavior or the acquisition of conditioned fear. This effect was also observed in over-conditioned mice environmentally manipulated to re-acquire fear extinction. Cumulatively, the effects of JZL184 appear to be partly due to augmentation of 2-AG signaling in the basolateral nucleus of the amygdala (BLA), as direct microinfusion of JZL184 into the BLA produced similar results. Moreover, we elucidate a short ~3-day temporal window during which 2-AG augmentation impairs extinction behavior, suggesting a preferential role for 2-AG-mediated eCB signaling in the modulation of short-term behavioral sequelae to acute traumatic stress exposure.

## Introduction

Emerging conceptualizations of several psychiatric disorders including addiction, schizophrenia and anxiety disorders highlight dysregulation of learning and memory processes as key contributors to disease pathogenesis. This is especially true for anxiety disorders including posttraumatic stress disorder (PTSD), where substantial preclinical and clinical data have identified specific abnormalities in fear-learning processes central to the pathophysiology of this illness.^1, 2, 3, 4, 5, 6, 7, 8, 9^

Exposure to traumatic stress initiates multiple neural processes, some of which are adaptive responses aimed at preventing harm. These include, for example, conditioned fear behavior triggered by cues or context associated with stress exposure. These acute fear responses facilitate harm avoidance and are adaptive in the short term. However, under pathological conditions these fear reactions can become generalized, which results in the expression of fear in the absence of explicit cues or contexts, and sensitized over time. Furthermore, neural processes such as extinction and habituation, which serve to reduce fear expression in response to cues or contexts previously associated with stressful events and that no longer accurately predict danger, are thought to be impaired. Thus, key pathophysiological substrates of PTSD include impairments in extinction learning and fear habituation, and exaggerated fear generalization and sensitization after traumatic stress exposure.^10^

For over a decade, increasing research has suggested a prominent role for endogenous cannabinoids (eCBs) in regulating fear-learning processes relevant to PTSD and other stress-related neuropsychiatric disorders.^11, 12, 13^ The eCBs are a class of bioactive lipids produced by neurons and glia in the central nervous system.^14^ 2-arachidonoylglycerol (2-AG) is the primary eCB that mediates eCB retrograde synaptic signaling at central synapses.^15^ 2-AG is synthesized post-synaptically via diacylglycerol lipase α (DAGLα) in an activity-dependent manner, and diffuses to presynaptic axon terminals where it interacts with type-1 cannabinoid receptors (CB1) to modulate neurotransmitter release.

Importantly, 2-AG signaling elements including CB1 receptors and DAGLα are highly expressed in the amygdala, prefrontal cortex and hippocampus, all regions implicated in PTSD pathophysiology.^16^ Moreover, compelling preclinical data indicate that mice deficient in CB1 receptors have impaired fear extinction and habituation, and represent a model for PTSD.^17^ Polymorphisms in CB1 gene are associated with PTSD,^18^ and exogenous activation of CB1 at low levels can facilitate fear extinction.^19, 20^ Consistent with this hypothesis, pharmacological enhancement of levels of the eCB anandamide (AEA) facilitates extinction of conditioned and acute fear,^19, 21^ and enhances extinction learning in a genetic model of impaired fear extinction.^22^ Together, these data suggest that AEA-mediated eCB signaling serves as a buffer to protect against the development of PTSD-like phenotypes in animal models.^23, 24^

Despite intriguing prior data, there had been little investigation of the specific role of 2-AG in the regulation of fear learning or extinction until recently, with studies implicating 2-AG both in facilitating and impairing fear extinction.^25, 26, 27^ Given these somewhat contradictory findings, the current study sought to clarify the effects of 2-AG signaling on various fear-learning processes, including short-term extinction learning, of relevance to stress-related neuropsychiatric disorders. Here we used a short-term extinction paradigm to test the hypothesis that pharmacological enhancement of 2-AG signaling in the brain may impair or facilitate active extinction learning of conditioned fear. Results showed that indirect pharmacological enhancement of 2-AG levels, via monoacylglycerol lipase (MAGL) inhibition, impair short-term fear extinction learning without affecting the acquisition or expression of conditioned fear. This effect was observed in a variety of behavioral paradigms, and required CB1 receptor availability. These data highlight potentially opposing roles of AEA and 2-AG on short-term fear extinction and suggest a more complex role for eCB signaling in the regulation of fear-learning processes than previously thought.

## Materials and methods

### Subjects and drug treatments

Male 30–35 g ICR mice (Harlan, Indianapolis, IN, USA) or 8–12-week-old C57BL/6 J (B6) (Jackson Laboratory, Bar Harbor, ME, USA) were used for fear extinction and microinfusion experiments, respectively. With the exception of intra-cranial infusion experiments (which necessitated individual housing to maintain cannula integrity), mice were housed five per cage in a temperature and humidity controlled housing facility under a 12-h light/dark cycle, with access to food and water *ad libitum*. At the time of testing, mice were treated with different doses of JZL184 (Vanderbilt University, Chemical Synthesis Core) dissolved in an 18:1:1 solution of saline, ethanol and kolliphor EL (Sigma Aldrich, St. Louis, MO, USA), or vehicle alone (18:1:1 solution of saline, ethanol and kolliphor EL) via i.p. injection 1h prior to behavioral testing. With the exception of a low-dose JZL184 pre-treatment (2 mg kg^−1^), all experiments included an 8 mg kg^−1^ dose. For some experiments, mice were treated with a combination of Rimonabant (Cayman Chemical, Ann Arbor, MI, USA) (3 mg kg^−1^) and JZL184 (8 mg kg^−1^) or Rimonabant alone (3 mg kg^−1^), via i.p. injection 1 h prior to behavioral testing. Timing and number of drug treatments are diagramed in each figure for clarity. PF-3845 (Cayman Chemical) was administered at 10 mg kg^−1^ for all experiments noted in figures. All studies were approved by the Vanderbilt University and NIAAA Institutional Animal Care and Use Committees, and were conducted in accordance with the National Institute of Health Guide for the Care and Use of Laboratory Animals.

### Conditioned fear- and short-term extinction protocols

A video fear-conditioning system and software (Med Associates, Burlington, VT, USA) were used to measure freezing in the ICR mice while in a conditioning chamber (dimensions: 30.5 × 24.1 × 21.0 cm), which was cleaned in between testing with Vimoba, a chlorine dioxide solution. Freezing was defined as no movement other than respiration.^28^ Mice were moved to a holding room adjacent to the test room and acclimated for 30 min (min) under 175–177 lux room lighting before testing.

For fear-conditioning procedures, mice were moved to the test room under the same conditions (175–177 lux) and then placed in the conditioning chamber for 30 s. After an initial one-half min of habituation (baseline), ICR mice were presented with six conditioned stimulus–unconditioned stimulus (CS–US) pairings (for example, tone–footshock pairings) separated by a 30-s interval. Each tone (80 dB, 3000 Hz) lasted 30 s. Mice were presented with the electric footshock at 0.7 mA during the last 2 s of each 30-s tone. The number of CS–US pairings and footshock intensity was used because ICR mice only present an appreciable increase in freezing behavior by the fourth CS–US presentation, demonstrating acquisition of conditioned fear learning (see Figures for illustrated examples). At the end of the fear-conditioning protocol, mice were placed in their home cages in a separate holding room before being returned to their housing facility.

The following day, mice were placed in the conditioning chamber with a white smooth floor contextual insert that was positioned over the grid floor and a white curved wall contextual insert (context B). Vanilla extract (McCormick, Sparks, MD, USA) was used as a distinctive olfactory cue in the conditioning chamber for the short-term extinction training. After 30 s of habituation in the chamber, mice were presented with 20 tones (80 dB, 3000 Hz) with durations of 30 s, each separated by a 30-s interval. After 20 min and 30 s, mice were returned to a holding room and their home cages.

The extinction protocol was repeated the following day using the same procedure and drug administration. For contextual fear conditioning, an identical paradigm was used except that all tones were omitted on all days of the experiment, and the extinction context was identical to that of the conditioning context. In overtraining experiments, mice were conditioned as described above for 5 consecutive days with the exact same procedure. The short-term extinction training procedure thereafter was identical to that described for the single conditioning experiments.

### Microinfusion and extinction retrieval

Under isoflurane anesthesia, 26 gauge bilateral guide cannulae (Plastics One, Roanoke, VA, USA) were targeted to the central lateral division of the central amygdala (CeA) (−1.30 mm anterior–posterior, ±2.80 mm mediolateral, −4.50 mm ventral to Bregma) or to the basolateral nucleus of the amygdala (BLA) (−1.30 mm anterior–posterior, ±3.17 mm mediolateral, −4.70 mm ventral to Bregma) of C57BL/6 J (B6) mice and held in place with dental cement. Mice were singly housed and given a 4–7-day post-surgery recovery period during which dummy cannulae were replaced daily to habituate mice to handling of cannulae and prevent blocking. JZL184 was bilaterally infused into the central lateral division and BLA at a dose of 0 or 5 μg μl^−1^ 30 min before extinction sessions on days 2 and 3. Infusions were performed at a volume of 0.2 μl per hemisphere over 4 min. Additional 2 min were allowed for diffusion of drug into brain tissue. Infusions were repeated on day 3.

For all microinfusion experiments, mice were fear conditioned in context A as previously described.^22^ On day 1, after a 180-s acclimation period, there were 3 × pairings (60–120-s interval) of the conditioned stimulus (CS; 30 s 80 dB, 3000 Hz tone) and the unconditioned stimulus (US; 2 s, 0.6 mA scrambled foot shock), in which the US was presented during the last 2 s of the CS. The session ended 120 s after the final CS–US pairing. This number of CS–US pairings and shock intensity was used because C57BL/6 J (B6) mice demonstrate an appreciable increase in freezing behavior by the 2nd CS–US presentation, demonstrating acquisition of conditioned fear learning.^22^ On days 2 and 3, extinction training was conducted as previously described.^22^ Extinction sessions were conducted in context B. On day 4, extinction retrieval was tested in context B following a 180-s acclimation period. On completion of testing, mice were infused with BODIPY TMR-X, SE (Thermo Fisher Scientific, Waltham, MA, USA), at 5 mm in 60% PBS and 40% DMSO (Invitrogen, Eugene, OR, USA) to examine the spread of each microinfusion volume. Mice were then perfused with 4% paraformaldehyde. Fixed brains were sectioned (50-μm thickness) on a vibrating microtome and stained with cresyl violet to verify the localization of the cannulae placements with the aid of a microscope. Mice that had more than minor spread of infusion volume into non-targeted amygdalar nuclei in both hemispheres were excluded from analysis.

### Statistical analysis

Time course freezing data were analyzed by two-way analysis of variance (ANOVA) factoring trial block and drug treatment. When significant effects of drug treatment were observed, *post hoc* analysis of total freezing time or percent freezing were analyzed by *t*-test. Total freezing time was calculated as the cumulative freezing time summated from each 30-s tone throughout the trial. All statistical analyses were conducted using Prism Graphpad 6 (Graphpad, San Diego, CA, USA). *P<*0.05 was considered significant throughout. F and *P*-values for significant effects of drug treatment by ANOVA are listed in figures above referenced graphs, as are *P*-values from *post hoc* analysis. For microinfusion experiments, percent freezing was calculated by hand-scoring freezing every 5 s. Average percent freezing for every five tones was binned and plotted, as depicted in figure axes. The effect of drug treatment on percent freezing during the first block (average of first five tones) of extinction retrieval was statistically analyzed using *t*-test. Sample sizes for all experiments are included in figure legends.

## Results

### JZL184 does not affect acquisition of conditioned fear

**Figure 1:**
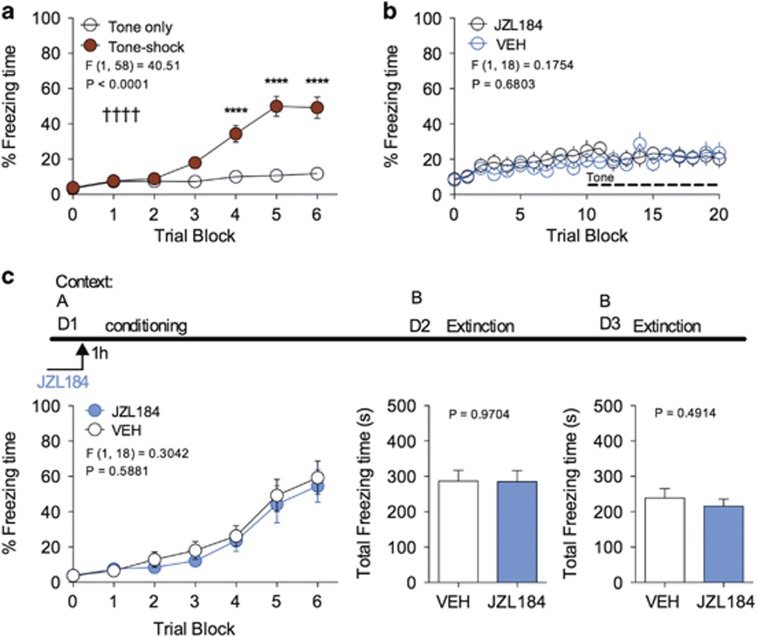
JZL184 does not affect acquisition of conditioned fear. (**a**) Effect of tone–shock (CS–US) pairings on freezing behavior in cued-conditioned fear learning. (**b**) Effects of JZL184 pre-treatment on freezing behavior in naive (non-conditioned) mice. (**c**) Fear conditioning and extinction schematic. Effect of JZL184 on freezing behavior during conditioning or short-term extinction sessions when administered prior to the conditioning session. ^†††^^†^*P<*0.0001 significant effect of conditioning treatment by two-way ANOVA. ****P<0.0001 significantly different from non-conditioned treatment by Sidak's multiple comparisons. Error bars represent s.e.m. (**a**) *n=*30 mice per group. (**b** and **c**) *n=*10 mice per group. ANOVA, analysis of variance; CS–US, conditioned stimulus–unconditioned stimulus.

To begin to test the role of 2-AG-mediated eCB signaling in the regulation of fear learning, we utilized the MAGL inhibitor JZL184 (8 mg kg^−1^) to systemically increase brain levels of 2-AG by blocking its degradation. This dose was chosen because it is sufficient to produce a 100–200% increase in brain 2-AG levels when administered i.p., that returns to baseline levels after 24 h, but does not cause locomotor suppression or CB1 receptor desensitization.^29, 30, 31^ Male ICR mice exposed to six shock–tone pairings exhibited significantly elevated freezing responses relative to mice presented with tone alone (Figure 1a), demonstrating that this line of mice begin to acquire freezing responses by the third and fourth CS–US presentation. Treatment with JZL184 in the absence of fear conditioning did not cause a significant freezing response on exposure to the conditioning chamber or on exposure to non-reinforced tone, relative to vehicle-treated mice (Figure 1b), suggesting JZL184 does not produce spontaneous unconditioned freezing.

In a separate experiment, pre-conditioning treatment with JZL184 did not affect the acquisition of freezing response to repeated shock–tone presentation (Figure 1c). Moreover, subsequent extinction training was not different between mice treated with JZL184 or vehicle prior to conditioning (Figure 1c, bar graphs). These data indicate that, at this dose, JZL184 does not cause non-specific freezing behavior in response to conditioning context or non-reinforced tone presentation, and does not affect the acquisition of freezing responses to conditioned fear stimuli.

### JZL184 impairs short-term extinction of auditory cue-conditioned fear

**Figure 2:**
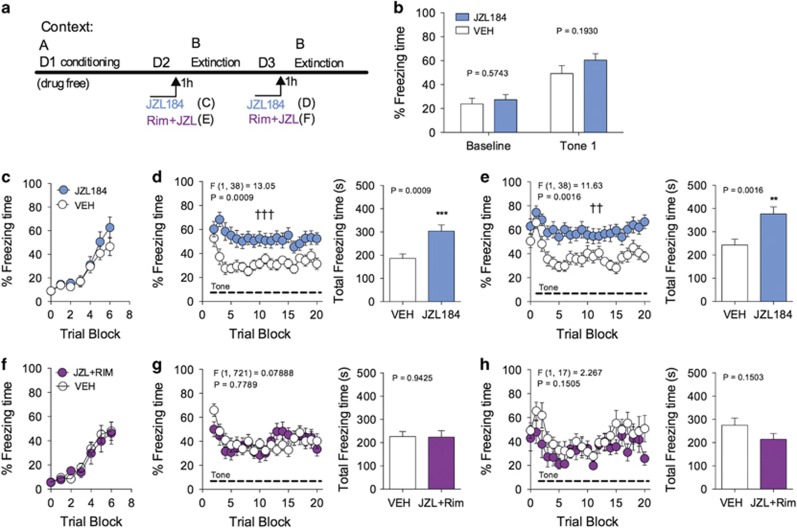
JZL184 impairs extinction of cue-conditioned freezing behavior. (**a**) Schematic diagram of the experimental paradigm and drug treatments. (**b**) Effects of JZL184 treatment prior to extinction on baseline freezing and conditioned freezing to the first tone presentation. (**c**) Freezing levels during acquisition of cue-conditioned fear (**d** and **e**) Effects of JZL184 on short-term extinction of conditioned freezing on days 1 and 2 of extinction training. (**f**) Freezing levels during acquisition of cue-conditioned fear. (**g** and **h**) Effects of Rimonabant and JZL184 co-administration on JZL184-induced short-term extinction impairment on days 1 and 2 of extinction. ^††^*P<*0.01, ^†††^*P<*0.001 significant effect of drug treatment by two-way ANOVA. **P<0.01, ***P<0.001 significantly different from vehicle treatment by unpaired two-tailed *t*-test (bar graphs). Error bars represent s.e.m. (**b**–**e**) *n=*20 mice per group. (**f**–**h**) *n=*10 mice per group. ANOVA, analysis of variance.

Given the prominent role of CB1 receptors in the regulation of the extinction of conditioned fear, we next examined the effects of JZL184 (8 mg kg^−1^) on short-term extinction learning of conditioned freezing using an auditory cue-conditioned fear paradigm (Figure 2a). ICR mice were conditioned in context A via presentation of six tone–shock pairings, and 24 and 48 h later presented with repeated intermittent tone presentations in a novel context B in the absence of any shocks (Figure 2a). Mice were treated with JZL184 or vehicle 1 h prior to each of the two short-term extinction sessions. Both treatment groups demonstrated conditioned fear learning prior to drug administration (Figure 2c). As expected, mice treated with vehicle that underwent short-term extinction training demonstrated a within-session decrease in conditioned freezing behavior with repeated intermittent presentation of the CS (Figures 2d and e), but still showed fear renewal exemplified by elevated freezing response to the first CS presentation 24 h later (during short-term extinction session 2. Figure 2e). Mice treated with JZL184 did not exhibit baseline-freezing differences when placed in context B 24 h after conditioning (Figure 2b), nor did they exhibit differences in freezing after the first tone presentation (Figure 2b). These data indicate that JZL184 does not affect contextual fear generalization or the initial retrieval of conditioned fear memory, as compared with vehicle treatment.

In contrast, mice treated with JZL184 exhibited a severe impairment in the short-term within-session extinction of conditioned freezing behavior during both extinction session 1 and 2 relative to vehicle-treated mice (Figures 2d and e). *Post hoc* analysis revealed that JZL184 treatment increased total freezing time during short-term extinction session 1 and 2 compared with vehicle treatment (Figures 2d and e, bar graphs). Interestingly, cue-conditioned impairment of short-term extinction was not seen when AEA levels were augmented using the fatty acid amide hydrolase (FAAH) inhibitor PF-3845 (10 mg kg^−1^), suggesting the impairment of short-term extinction is unique to 2-AG signaling (Supplementary Figures S1b and c). Previous studies have demonstrated bi-phasic effects of cannabinoids on fear extinction with low doses facilitating extinction and high doses impairing extinction,^27, 32^ suggesting JZL184 could potentially exert bi-phasic effects on short-term extinction; however, a low dose of JZL184 (2 mg kg^−1^) did not cause impairment or enhancement of short-term fear extinction (Supplementary Figures S2b and c).

Since pharmacological inhibition of MAGL increases a class of MAGs, of which 2-AG is only one, and 2-AG is the only MAG with activity at CB receptors, we wanted to confirm that the effects of JZL184 on fear during short-term extinction training were mediated via augmentation of 2-AG signaling at CB1 receptors. Thus, we determined the effects of the CB1 receptor antagonist Rimonabant (3 mg kg^−1^) on JZL184-induced short-term extinction impairment. Mice from the vehicle-treated group or mice co-treated with Rimonabant and JZL184 demonstrated similar conditioned fear learning prior to drug administration (Figure 2f). Mice co-treated with Rimonabant and JZL184 did not exhibit any significantly elevated freezing during fear extinction training in session 1 or 2, relative to vehicle-treated mice (Figures 2g and h). Importantly, when administered alone, low-dose Rimonabant (3 mg kg^−1^) had no significant effect on freezing within short-term extinction sessions, and only significantly impaired extinction at a higher dose (10 mg kg^−1^) (Supplementary Figure S3c). These data confirm that the extinction-impairing effects of JZL184 are mediated via activation of CB1 receptors.^27^

### JZL184 prevents contextual facilitation of short-term extinction after overconditioning

**Figure 3:**
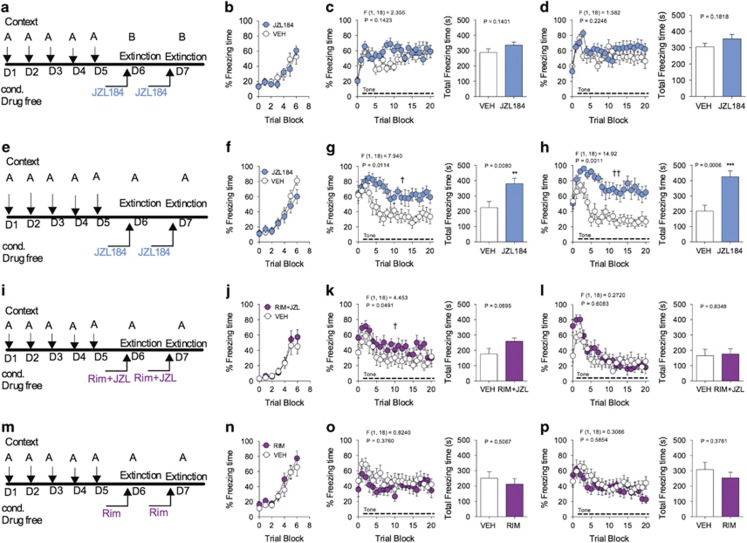
Contextual facilitation of short-term fear extinction after overconditioning is impaired by JZL184. (**a**) Schematic of JZL184 administration paradigm during short-term extinction of cue-conditioned overtraining. (**b**) Freezing levels during acquisition of cue-conditioned fear. (**c**) Effects of JZL184 on freezing during short-term extinction session 1, following cue-conditioned overtraining. (**d**) Effects of JZL184 on freezing during short-term extinction session 2, following cue-conditioned overtraining. (**e**) Schematic of JZL184 administration paradigm during contextual facilitation of cue-conditioned overtraining. (**f**) Freezing levels during acquisition of cue-conditioned fear. (**g**) Effects of JZL184 on freezing during facilitated contextual extinction session 1, following cue-conditioned overtraining. (**h**) Effects of JZL184 on freezing during facilitated contextual extinction session 2, following cue-conditioned overtraining. (**i**) Schematic of JZL184 and Rimonabant co-administration paradigm during contextual facilitation of cue-conditioned overtraining. (**j**) Freezing levels during acquisition of cue-conditioned fear. (**k**) Effects of JZL184 and Rimonabant co-administration on freezing during facilitated contextual extinction session 1, following cue-conditioned overtraining. (**l**) Effects of JZL184 and Rimonabant co-administration on freezing during facilitated contextual extinction session 2, following cue-conditioned overtraining. (**m**) Schematic of Rimonabant administration paradigm during contextual facilitation of cue-conditioned overtraining. (**n**) Freezing levels during acquisition of cue-conditioned fear. (**o**) Effects of Rimonabant on freezing during facilitated contextual extinction session 1, following cue-conditioned overtraining. (**p**) Effects of Rimonabant on freezing during facilitated contextual extinction session 2, following cue-conditioned overtraining. ^†^*P<*0.05, ^††^*P<*0.01 significant effect of drug treatment by two-way ANOVA. **P<0.01, ***P<0.001 significantly different from vehicle treatment by unpaired two-tailed *t*-test (bar graphs). Error bars represent s.e.m. *n=*10 mice per group for each experiment. ANOVA, analysis of variance.

To further explore the environmental determinants of JZL184-induced impairment in extinction, we examined the effects of JZL184 on short-term extinction learning of cue-conditioned freezing behavior after overtraining. Mice were conditioned for 5 consecutive days, followed 24 and 48 h later by extinction sessions (Figure 3a). Both treatment groups demonstrated similar conditioned fear learning on the first day of conditioning (Figure 3b). Mice were treated with JZL184 or vehicle 1 h prior to both extinction sessions. As expected, overtraining results in a resistance to within-session extinction of cue-conditioned freezing behavior during both short-term extinction sessions (Figures 3c and d). Surprisingly, JZL184 treatment did not significantly affect extinction learning relative to vehicle treatment after overconditioning (Figures 3c and d). *Post hoc* analysis revealed no significant effect of JZL184 on the total time spent freezing (Figures 3c and d, bar graphs).

Since the lack of impairment of JZL184 on short-term extinction of cue-conditioned overtraining may be due to a ceiling effect, we next examined whether JZL184 would impair contextual facilitation of extinction in over-trained mice. We hypothesized that the combined presentation of auditory cues and contextual stimuli during short-term extinction sessions would serve to promote within-session extinction of freezing behavior in over-trained subjects. Mice received overtraining in context A and were then placed back into context A 24 and 48 h later for extinction sessions in the presence of auditory cues (Figure 3e). Both groups demonstrated conditioned fear learning on the first day of conditioning (Figure 3f). As expected, vehicle-treated mice placed back into the same context in the presence of auditory cues demonstrated facilitation of short-term extinction (Figures 3g and h). Notably, JZL184-treated mice had significantly elevated freezing behavior as compared with vehicle-treated mice (Figures 3g and h). *Post hoc* analysis revealed significantly greater total freezing time in JZL184-treated mice as compared with vehicle-treated mice (Figures 3g and h, bar graphs). These data suggest that JZL184 prevents contextual facilitation of short-term extinction learning following overtraining. Importantly, this effect was blocked with the co-administration of JZL184 and Rimonabant (3 mg kg^−1^) (Figures 3i and l). *Post hoc* analysis did not reveal a significant difference in total freezing time between JZL184 and vehicle-treated mice (Figures 3k and l, bar graphs), suggesting that JZL184 impairs contextual facilitation of short-term extinction in a CB1 receptor-dependent manner. The ability of Rimonabant to block the effects of JZL184 is not likely due to off target action, as Rimonabant treatment at this dosage did not significantly alter freezing behavior in extinction sessions when administered alone to over-trained mice (Figures 3m and p).

### A narrow temporal window limits JZL184 effects on short-term fear extinction

**Figure 4:**
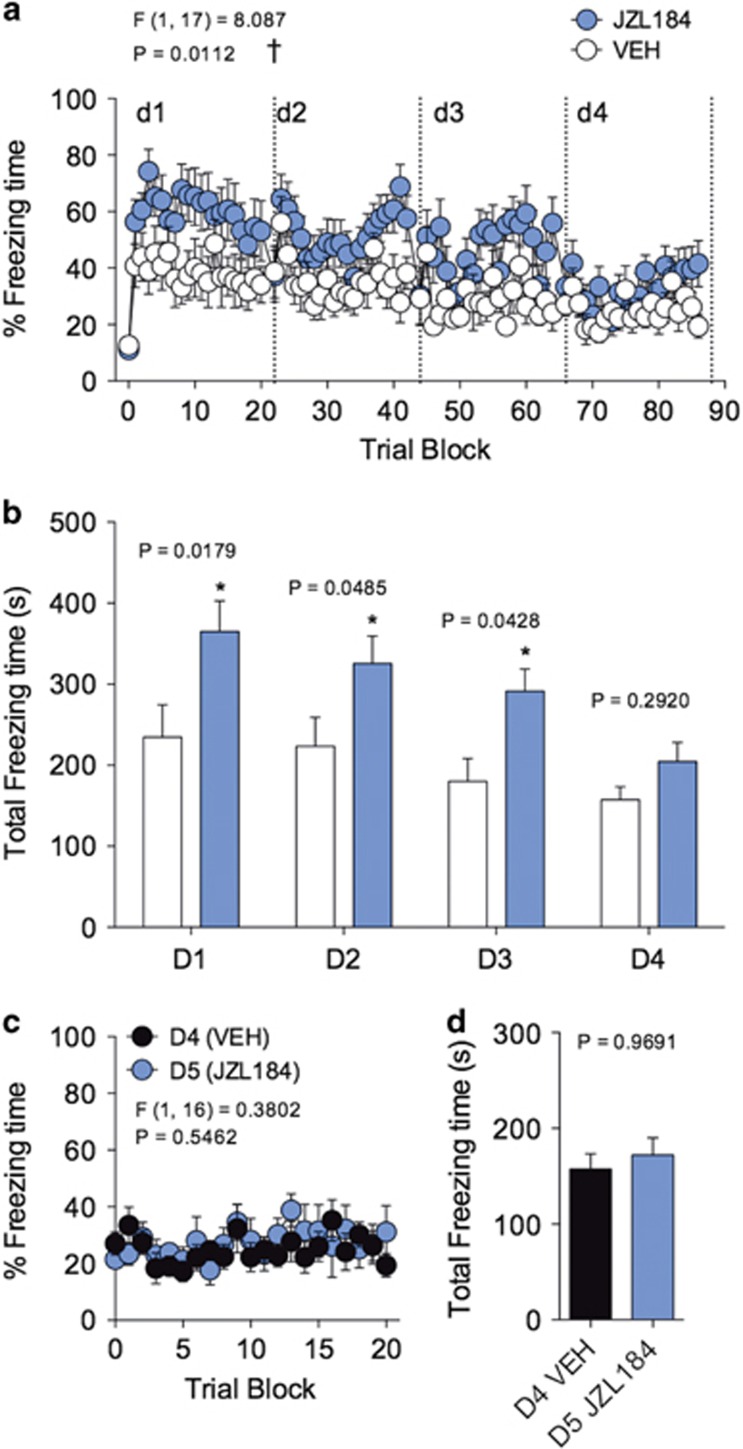
JZL184 effects on short-term fear extinction are limited by a narrow temporal window. (**a**) Effects of JZL184 on freezing during 4 days of short-term extinction training. (**b**) Effects of JZL184 on total time freezing during 4 days of short-term extinction training. (**c**) Effects of JZL184 on freezing of drug-naive mice on day 5, compared with freezing behavior on day 4. (**d**) Effects of JZL184 on total freezing time of drug-naive mice on day 5, compared with freezing behavior on day 4. ^†^*P<*0.05 significant effect of drug treatment by two-way ANOVA. *P<0.05 significantly different from vehicle treatment by unpaired two-tailed *t*-test (bar graphs). Error bars represent s.e.m. *n=*10 mice per group. ANOVA, analysis of variance.

Since administration of JZL184 impaired cued extinction during each short-term extinction session, we sought to determine whether subjects were still capable of completely extinguishing conditioned freezing behavior over repeated short-term extinction sessions under the influence of JZL184. Daily short-term extinction training led to long-term extinction of conditioned freezing behavior (Figure 4a). Mice treated with JZL184 demonstrated long-term extinction of freezing behavior to levels seen in vehicle-treated control mice after 4 days of short-term extinction sessions (Figures 4a and b). Total time freezing was significantly elevated for the first 3 days of short-term extinction training in JZL184-treated mice as compared with vehicle-treated mice, but was not significantly different between JZL184 and vehicle-treated mice by day 4 (Figure 4b). These data suggest that there is a narrow 3-day window in which JZL184 is capable of impairing short-term extinction learning, and that given enough short-term extinction training sessions JZL184-treated mice will extinguish conditioned freezing responses.

To assess whether the time window of JZL184 effects on impairing short-term extinction is due to drug tolerance, we administered JZL184 to drug-naive subjects that had received vehicle treatment over the first 4 days of extinction training. On day 5 of extinction training, drug naive subjects that received JZL184 did not show significantly elevated levels of freezing behavior relative to day 4 (Figures 4c and d), suggesting that the temporal window that limits JZL184 effects on short-term extinction impairment is not due to drug tolerance resulting from repeated drug administration.

### JZL184 enhances fear sensitization

**Figure 5:**
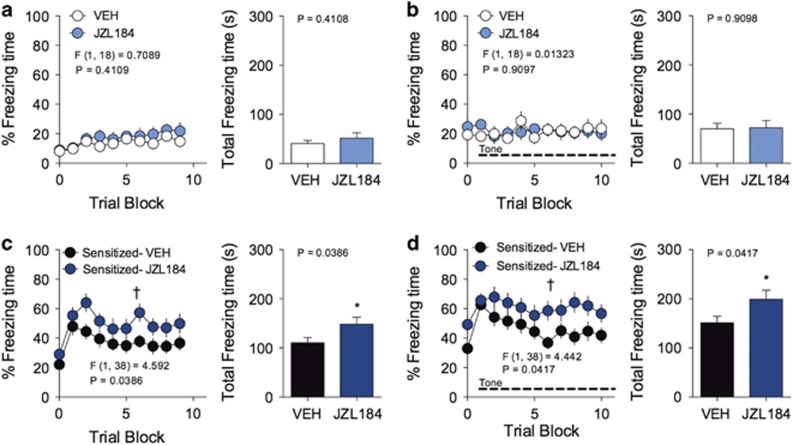
JZL184 enhances fear sensitization. (**a**) Effects of JZL184 on freezing of non-conditioned mice placed in a novel context. (**b**) Effects of JZL184 on freezing of non-conditioned mice placed in a novel context during the presentation of auditory tones. (**c**) Effects of JZL184 on freezing of contextual-conditioned mice placed in a novel context. (**d**) Effects of JZL184 on freezing of contextual-conditioned mice placed in a novel context with the presentation of auditory tones. ^†^*P<*0.05 significant effect of drug treatment by two-way ANOVA. *P<0.05 significantly different from vehicle treatment by unpaired two-tailed *t*-test (bar graphs). Error bars represent s.e.m. *n=*10 mice per group. ANOVA, analysis of variance.

In addition to fear learning, eCBs play a large role in regulating generalized anxiety behavior and physiological responses to stress exposure.^12, 15, 33, 34, 35^ Since JZL184 impairs short-term extinction, we next asked whether JZL184 mediates its effects through enhancement of sensitization to stressful or innate unconditioned freezing-inducing stimuli. Mice were conditioned in context A in the absence of auditory cues to induce contextual fear learning. Mice were then injected 24 h later with either vehicle or JZL184 1 h prior to being placed into context B for a fear sensitization trial. The fear sensitization trial consisted of 10 min without auditory tones (Figures 5a and c), followed by 10 min of auditory tone presentation (Figures 5b and d). Exposure to context B serves as a novel environment for the fear conditioned mice, where fear sensitization to contextual (no tone) or unconditioned auditory (with tone) stimuli can be measured by recording freezing behavior. As expected, mice that did not receive contextual conditioning in context A did not demonstrate elevated freezing behavior in context B when treated with vehicle or JZL184 (Figures 5a and b). However, in mice receiving non-reinforced footshocks in context A, JZL184-treated mice demonstrated significantly greater freezing compared with vehicle-treated mice during exposure to context B in the presence or absence of auditory tones (Figures 5c and d). These data demonstrate that JZL184 enhances fear sensitization to both novel contextual and unconditioned auditory stimuli.

### JZL184 infusion into the BLA enhances retention of learned fear

**Figure 6:**
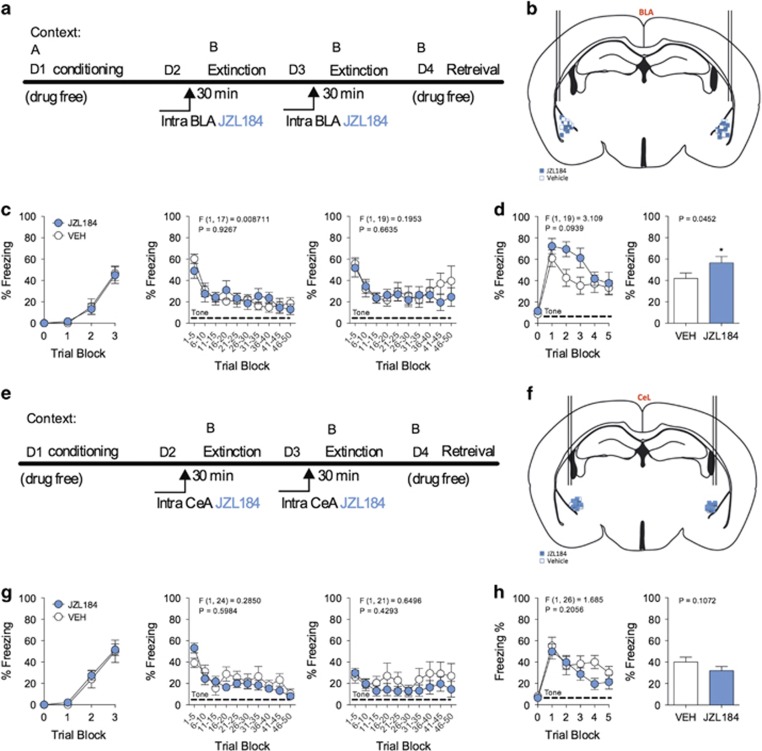
JZL184 microinfusion into the BLA enhances retention of cue-conditioned fear. (**a**) Schematic of JZL184 BLA-specific microinfusion paradigm and drug-free retrieval. (**b**) Coronal representation of BLA microinjection targeting for each subject determined by *post hoc* analysis of injection sites. (**c**) Effects of JZL184 microinfusion into the BLA on freezing during cue-conditioning and short-term extinction sessions. (**d**) Effects of JZL184 on freezing during a drug-free retrieval test. (**e**) Schematic of JZL184 CeA-specific microinfusion paradigm and drug-free retrieval. (**f**) Coronal representation of CeA microinjection targeting for each subject determined by *post hoc* analysis of injection sites. (**g**) Effects of JZL184 microinfusion into the CeA on freezing during cue-conditioning and short-term extinction sessions. (**h**) Effects of JZL184 on freezing during a drug-free retrieval test. *P<0.05 significantly different from vehicle treatment by unpaired one-tailed *t*-test (bar graphs). Error bars represent s.e.m. (**a**–**d**) *n=*12 mice for JZL184 group, and *n=*9 mice for vehicle group. (**e–h**) *n=*19 mice for JZL184 group, and *n=*10 mice for vehicle group. BLA, basolateral nucleus of the amygdala; CeA, central amygdala.

Given the prevalence of eCB synthetic machinery and signaling within the amygdala^14, 36, 37, 38^ and the amygdala's significant role in fear-learning processes,^39, 40, 41, 42, 43, 44^ we tested the hypothesis that JZL184 impairment of short-term extinction is due to enhancement of 2-AG signaling in the amygdala. To target JZL184 to the amygdala, we performed surgical craniotomy to insert bilateral cannulae above the BLA or CeA in C57BL/6 J (B6) mice (Figures 6b and f). This strain of mice was used because they demonstrate quick and robust fear conditioned learning to a small number of CS–US presentations,^22, 45^ and thus serve as a good model to limit potential confounds of surgical craniotomy and recovery on producing deficits in fear learning. Following recovery from surgery, mice were placed in context A and underwent cued-conditioning, as described above. We then performed *in vivo* bilateral microinfusion of JZL184 or vehicle into either the BLA or CeA 30 min prior to extinction sessions in context B on days 2 and 3 (Figures 6a and e). On day 4, we re-exposed mice to context B in the absence of drug to assess the retention of learned fear in a drug-free environment following short-term extinction training (Figures 6a and e).

Microinfusion of JZL184 into the BLA or CeA did not have a significant effect on freezing behavior during each short-term extinction session as compared with vehicle (Figures 6c and g). However, microinfusion of JZL184 into the BLA caused a significant increase in freezing during the retention test (Figure 6d). This effect was restricted to 2-AG augmentation in the BLA, as JZL184 microinfusion into the CeA did not significantly affect freezing in comparison to vehicle (Figure 6h). These data suggest that 2-AG augmentation in the BLA enhances the retention of cue-conditioned fear at least 24 h following short-term extinction.

## Discussion

In this study, we investigated global and region-specific effects of 2-AG augmentation on short-term extinction of conditioned fear. We and others have recently shown that 2-AG augmentation is anxiolytic in models of unconditioned fear learning and stress exposure.^31, 46, 47, 48^ To examine the role of 2-AG augmentation on the extinction of conditioned fear learning and extinction, we utilized a short-term extinction training protocol that promotes within-session extinction of fear behavior but allows mice to retain aversive fear memory of the CS the following day.

Using this paradigm, we found that global 2-AG augmentation using systemic administration of JZL184 prior to extinction training sessions was capable of impairing short-term extinction learning as measured by within-session freezing behavior. Our data also support the notion that impairment of short-term extinction learning by JZL184 is partly due to increased fear sensitization, as exposure to a new context and novel tone presentations in previously shocked mice resulted in elevated freezing after JZL184 relative to vehicle treatment. We also find that the effects of JZL184 on impairing short-term extinction learning are mediated by CB1 receptors. These data are consistent with a recent report, demonstrating that impairment in fear extinction after JZL184 administration is dependent on CB1 receptors expressed on GABAergic, but not glutamatergic forebrain neurons.^27^ In accordance with this hypothesis, global knockout of CB1 receptors or selective knockout of CB1 receptors from glutamatergic neurons has been shown to demonstrate a shift to more passive fear coping strategies, such as freezing, rather than active fear coping strategies, such as escape behavior.^32^ Alternatively, selective knockout of CB1 receptors from GABAergic neurons results in more active fear coping strategies.^32^ Cumulatively, these data suggest that CB1 signaling on GABAergic synapses may be important in the regulation of passive fear coping strategies, such as freezing in response to a threatening or traumatic stimulus. Therefore, another interpretation of our results is that 2-AG augmentation with JZL184 facilitates passive fear coping strategies, and thus enhances freezing behavior in response to a CS associated with a learned threat.

Interestingly, JZL184 did not effect freezing behavior in the conditioning session or subsequent short-term extinction sessions when administered prior to conditioning, which suggests that JZL184 does not affect the acquisition of fear learning. However, the lack of an effect of JZL184 on fear acquisition could also be due to our use of a relatively high number of presentations of CS–US pairings (6) during conditioning, resulting in a ceiling effect. Although this paradigm, and our use of 5-day overtraining, may be more useful for accurately reflecting pathological conditions of high fear and anxiety, it may also mask effects of JZL184 on the acquisition of fear learning. Indeed, JZL184 has recently been shown to increase freezing behavior during fear acquisition and on a retention test when fewer CS–US parings (3) are presented during conditioning;^49^ although we cannot rule out that this finding is due to the use of a different mouse strain. Further studies will be required to determine the role of 2-AG signaling in the acquisition of conditioned freezing responses.

Given that JZL184 did not significantly effect freezing when administered prior to conditioning, but enhanced freezing when administered prior to short-term extinction training, it is likely that the effect of 2-AG on fear-learning mechanisms is dependent on the timing of administration. Consistent with this hypothesis, we found that the impairment in short-term fear extinction by JZL184 only lasted up to 3 days, further supporting the idea of a narrow temporal window in which JZL184 is capable of producing within-session impairment of freezing behavior. Importantly, when vehicle-treated mice were exposed to multiple short-term extinction sessions, and subsequently treated with JZL184 prior to the 5th extinction session, no impairment was observed. Therefore, although 2-AG augmentation shortly after conditioning appears to worsen the adverse effects of traumatic stress, this effect is short lived, and may be balanced by sustained beneficial effects on unconditioned anxiety-like behaviors and/or fear generalization.

Although global increases in 2-AG signaling from systemic administration could synergistically contribute to impairment of extinction by acting on multiple brain structures, we hypothesized that 2-AG augmentation in amygdalar nuclei heavily implicated in mechanisms of fear learning are the most likely candidates for the effects of JZL184. Contextual fear learning is generally regulated by the hippocampus, whereas the expression of auditory-cued fear learning is primarily regulated by multiple amygdalar nuclei.^50^ However, interconnectivity between these regions has also been shown to regulate the formation of fear memories and anxiety phenotypes,^51, 52^ suggesting that 2-AG augmentation could effect behavior by suppressing specific circuitry between brain structures rather than exerting actions on one brain structure over another. Since we did not find a large effect of post-conditioning JZL184 treatment on impairment of contextual extinction (data not shown), which is consistent with previous findings that JZL184 does not effect the memory of contextual fear learning,^25^ we decided to focus our region-specific microinfusions to the nuclei of the amygdala that play a significant role in the acquisition, expression and retention of auditory cue-conditioned fear.^53, 54^ Surprisingly, we found that JZL184 microinfusion into the BLA enhances the retention of learned fear in a drug-free environment 24 h after short-term extinction training. This result suggests that the impairment of within-session extinction learning in our previous experiments could, at least in part, be due to 2-AG augmentation in the BLA contributing to the retention of learned fear. However, the finding that JZL184 microinfusion into the BLA did not effect within-session freezing during short-term extinction sessions could be due to differences in mouse strains or extinction training procedures used in this study. We cannot rule out the possibility that 2-AG production, degradation or CB1 receptor availability in the amygdala differs significantly between ICR and C57BL/6 J mice.

Consistent with our data suggesting a contributing role of the BLA in JZL184-induced extinction impairment, MAGL expression and CB1 expression have been noted at specific cholecystokinin-expressing invaginating GABAergic synapses in the BLA that are presynaptic to DAGLα-expressing pyramidal neurons, whereas MAGL appears mostly absent at BLA glutamatergic synapses that co-localize with DAGLα and CB1.^38^ These neuroanatomical data suggest that JZL184 augmentation of 2-AG levels may preference signaling at CB1-expressing terminals of specific GABAergic synapses in the BLA over glutamatergic synapses. The localization of molecular eCB machinery at these synapses is consistent with behavioral findings of JZL184 effects on fear expression. For example, JZL184-induced impairment in fear extinction is absent in mice where CB1 was selectively knocked out in GABAergic neurons.^27^ Cumulatively, these findings suggest cholecystokinin+ GABAergic signaling within the BLA may be important for extinction learning, and 2-AG may serve to impair extinction by reducing GABAergic tone onto pyramidal neurons actively involved in cue-conditioned memory traces.

Related to the above point, the facilitation of fear extinction by specific selective serotonin reuptake inhibitor can be partly explained by previously unrecognized effects on eCB signaling. For example, fluoxetine's ability to promote extinction retention requires AEA signaling at CB1 receptors in the BLA.^55^ Interestingly, this effect of AEA was associated with enhanced tonic CB1 activity at GABAergic synapses in the BLA, calling into question how pharmacologic augmentation of 2-AG and AEA in the BLA can result in opposing effects on the retention of fear extinction if both act at the same GABAergic CB1 receptors. One explanation is that the position of AEA and 2-AG synthetic machinery or magnitude of AEA and 2-AG production differs between physiologically defined pyramidal cells in the BLA. Indeed, *in vivo* recordings and optogenetic studies have demonstrated different populations of pyramidal neurons in the BLA that are important for fear memory formation and fear memory extinction.^56^ These pyramidal cells in the BLA have been classified as either ‘fear' neurons or ‘extinction' neurons depending on their increase in activity to the presentation of a CS during conditioning/retention sessions or extinction sessions, respectively. Accordingly, AEA synthesis could occur most prevalently at ‘extinction' neurons, whereas 2-AG synthesis could occur most prevalently at ‘fear' neurons. In this scenario, pharmacologic augmentation of AEA could result in enhanced tonic AEA signaling at GABAergic synapses onto extinction neurons, resulting in an overall increase in extinction neuron activity and a behavioral inhibition of conditioned freezing during the retention test. Likewise, pharmacologic augmentation of 2-AG would result in elevated activity-dependent 2-AG signaling at GABAergic synapses onto fear neurons, resulting in an increase in fear neuron activity and corresponding increase in freezing behavior during the retention test. Recent advances in identifying functionally defined cell types in the BLA by the chemical markers they express suggests the feasibility of testing this hypothesis in the future. Extinction neurons may have already been identified, primarily, by their expression of the Thy1 cell surface antigen.^57^ Testing the capacity of AEA and 2-AG signaling at GABAergic and glutamatergic synapses onto these classes of neurons in the BLA could help clarify the differences in AEA and 2-AG augmentation on the retention of extinction learning.

Even though there are multiple possible explanations for the effects of JZL184 on fear learning, our results and those of Llorente-Berzal^27^ both contrast recent work demonstrating constitutive DAGLα deletion, which reduces 2-AG levels, also impairs fear extinction.^26^ Our data also contrast multiple findings indicating JZL184 decreases unconditioned anxiety and stress responses, as described above. Therefore, it appears pharmacological augmentation of 2-AG signaling can impair short-term fear extinction or enhance passive freezing behavior, but reduce unconditioned generalized anxiety behaviors, emphasizing the complexity and construct-specific effects of eCBs on emotional behaviors.

The apparently disparate effects seen with systemic JZL184 administration on conditioned fear behaviors and unconditioned anxiety behaviors also warrants some discussion. One explanation for this apparent difference is based on the hypothesis that conditioned fear and unconditioned anxiety are regulated by different neural circuits. Although brain regions shown to be important for producing conditioned fear and unconditioned anxiety partially overlap, growing evidence suggests, even within individual brain structures, that specific circuitries are capable of bi-directionally controlling the expression of these distinct processes.^58^ This is particularly true for amygdalar nuclei, where the CeA contains an inhibitory microcircuit capable of gating the expression of conditioned fear through disinhibition of CeA output following excitation from glutamatergic afferents from thalamic nuclei or the lateral amygdala onto specific cell types;^59, 60^ yet, glutamatergic input from the BLA to the CeA can also serve to be anxiolytic, while activation of the BLA alone can be anxiogenic.^61^ Therefore, even within a functionally or anatomically defined brain structure, such as the amygdala, circuits that modulate conditioned fear and unconditioned anxiety can be non-overlapping and specific to separate populations of neurons.

It is possible that 2-AG augmentation exerts opposing effects on fear and anxiety behaviors due to its action on specific glutamatergic circuits that differentially promote or inhibit the expression of fear and anxiety phenotypes. At a molecular level, research supports the role of on-demand synthesis and release of 2-AG for inhibition at putative glutamatergic synapses,^62, 63^ suggesting MAGL inhibition using JZL184 may prolong CB1-mediated inhibition of glutamate release in an activity-dependent manner via heterosynaptic spillover and sustained presence of 2-AG within excitatory synapses due to lack of overall degradation. 2-AG signaling is also prominent at glutamatergic synapses in the amygdala,^37, 64^ suggesting that JZL184 could exert its effects by elevating 2-AG signaling at excitatory synapses in addition to inhibitory synapses. It is not unreasonable to hypothesize that the activity of different circuits, which converge on amygdalar nuclei, are only recruited during specific types of environmental stress exposure. Since 2-AG has an overall effect of dampening action potential-dependent release of both GABA and glutamate, augmentation of 2-AG signaling in non-stressed mice may serve to depress the excitatory synapses of circuits that promote anxiety expression in an activity-dependent manner. This could happen specifically during exposure to novel environments, where avoidance of predation is equally important to exploration, approach behavior and foraging for new resources, and mounting rapid or autonomic defensive responses characteristic of fear behavior does not serve a necessary adaptive advantage. Similarly, 2-AG augmentation may depress excitatory drive onto specific circuits that signal to inhibit fear memory expression, amounting in an overall average increase in conditioned freezing responses in situations when circuitry regulating more generalized apprehensive behaviors is not normally recruited. Because our results indicate 2-AG augmentation only serves to sensitize mice to unconditioned stimuli following footshock exposure, it is possible that elevated 2-AG signaling can acutely override or mask the anxiolytic properties of JZL184 when an animal is exposed to a highly salient threat.

Despite converging evidence for a role of eCBs in fear-learning processes, there is a critical gap in our understanding of how the two main eCBs, AEA and 2-AG, could redundantly or differentially modulate extinction of conditioned fear. Pharmacological augmentation of AEA has proved to be a promising approach for facilitating extinction of learned fear in previous studies,^11^ suggesting perturbations of eCB signaling may have therapeutic efficacy in treating stress-related psychiatric disorders. However, here we demonstrate that augmentation of 2-AG levels using the MAGL inhibitor JZL184 paradoxically impairs short-term fear extinction during a narrow time window after conditioning. These effects are dependent on canonical eCB signaling at CB1 receptors, raising the question of how such a ubiquitous signaling mechanism in the brain is capable of differential behavioral effects. Future research into brain-region/circuit-specific and cell-type-specific components of eCB machinery may yield novel insights into how pharmacological manipulations of AEA and 2-AG produce opposing behavioral results, and open new avenues for identifying therapeutic targets in the treatment of fear- and anxiety-related psychiatric disorders.
