# Supplementary figures and images for: 2-arachidonoylglycerol signaling impairs short-term fear extinction
Source: Transl Psychiatry. 2016 Mar 1;6(3):e749–. doi: 10.1038/tp.2016.26 (PMC4872450; doi:10.1038/tp.2016.26)

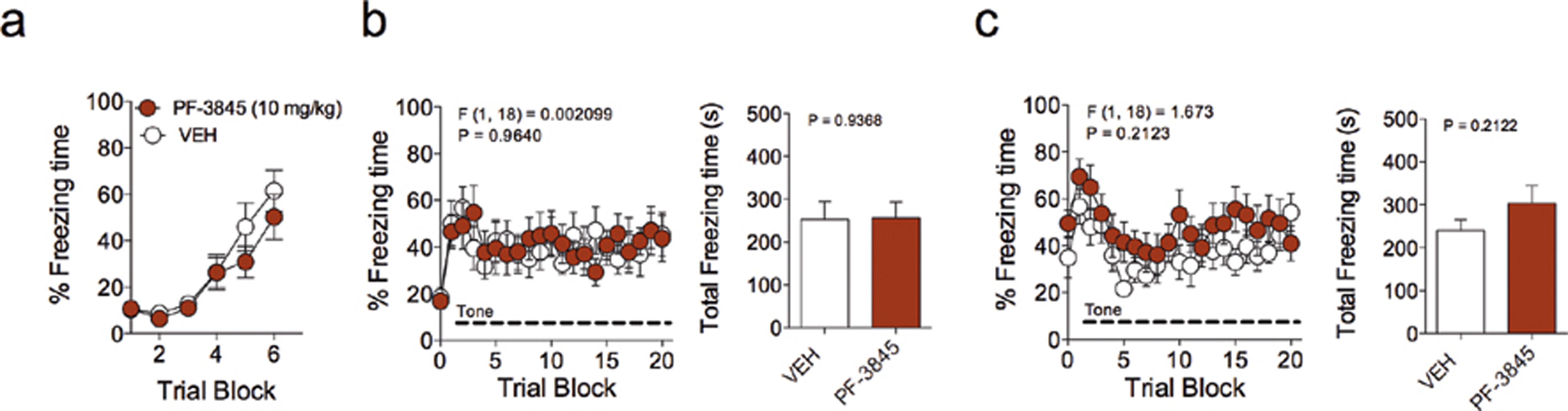

Supplement: Supplementary Figure S1 [file tp201626x1.tif]

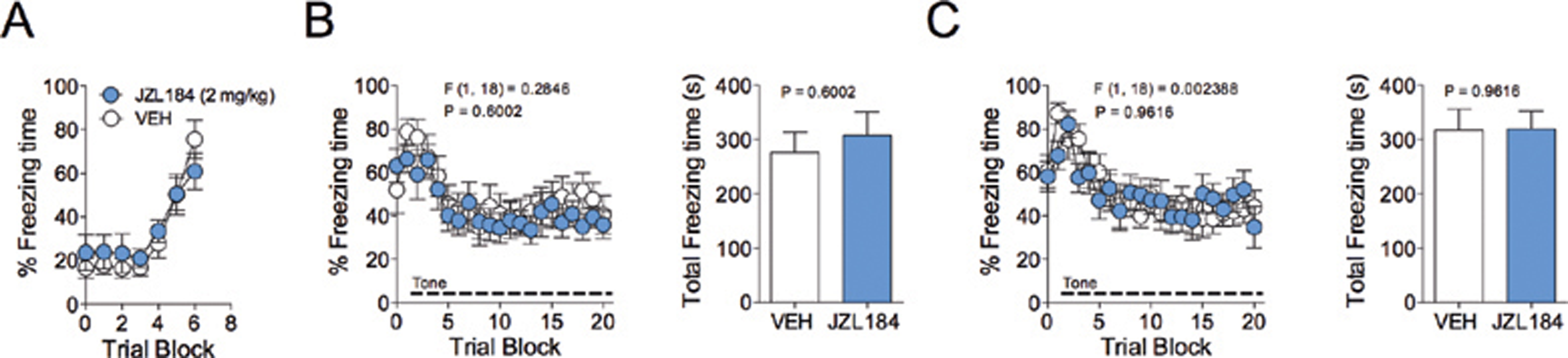

Supplement: Supplementary Figure S2 [file tp201626x2.tif]

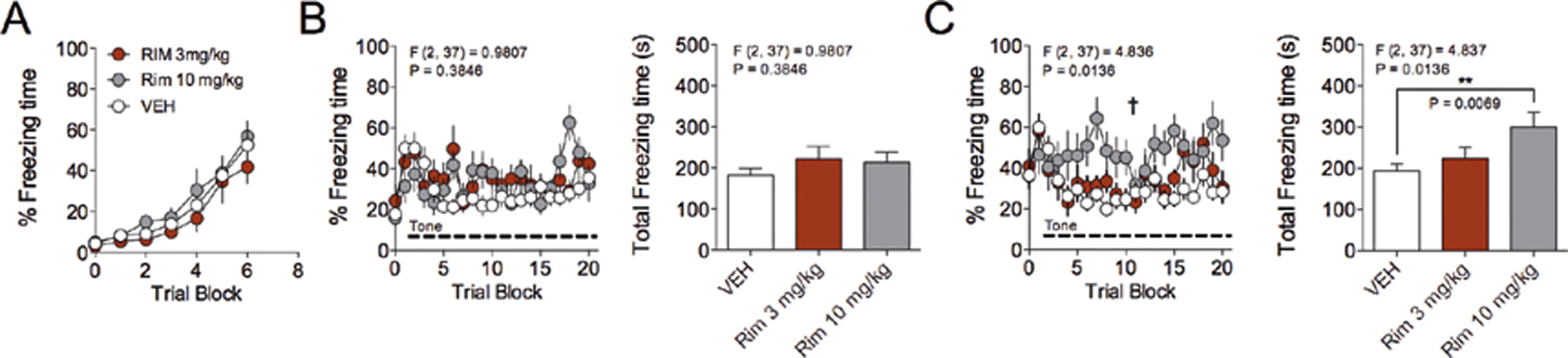

Supplement: Supplementary Figure S3 [file tp201626x3.tif]
