# Supplementary material for: 2-arachidonoylglycerol signaling impairs short-term fear extinction
Source: Transl Psychiatry. 2016 Mar 1;6(3):e749–. doi: 10.1038/tp.2016.26 (PMC4872450; doi:10.1038/tp.2016.26)
Supplement: Supplementary Figure Legends [file tp201626x4.doc]

**Supplementary Figure Legends**

**Supplement Figure 1. PF-3845 does not impair short-term extinction of cue-conditioned fear. (a)** Freezing levels during acquisition of cue-conditioned fear. **(b)** Effects of PF-3845 on freezing during short-term extinction session 1. **(c)** Effects of PF-3845 on freezing during short-term extinction session 2. n=10 mice per group.

**Supplement Figure 2. Low-dose JZL184 does not impair short-term extinction of cue-conditioned fear. (a)** Freezing levels during acquisition of cue-conditioned fear. **(b)** Effects of JZL184 (2mg/kg) on freezing during short-term extinction session 1. **(c)** Effects of JZL184 (2mg/kg) on freezing during short-term extinction session 2. n=10 mice per group.

**Supplement Figure 3. High-dose Rimonabant impairs short-term extinction of cue-conditioned fear. (a)** Freezing levels during acquisition of cue-conditioned fear. **(b)** Effects of low-dose (3mg/kg) and high-dose (10mg/kg) Rimonabant on freezing during short-term extinction session 1. **(c)** Effects of low-dose (3mg/kg) and high-dose (10mg/kg) Rimonabant on freezing during short-term extinction session 2. † p < 0.05 significant effect of drug treatment by two-way ANOVA. ** p < 0.05 significant effect of drug treatment by one-way ANOVA. n=10 mice per group.
